# Musculoskeletal Features without Ataxia Associated with a Novel de novo Mutation in *KCNA1* Impairing the Voltage Sensitivity of Kv1.1 Channel

**DOI:** 10.3390/biomedicines9010075

**Published:** 2021-01-14

**Authors:** Paola Imbrici, Andrea Accogli, Rikard Blunck, Concetta Altamura, Michele Iacomino, Maria Cristina D’Adamo, Anna Allegri, Marina Pedemonte, Noemi Brolatti, Stella Vari, Matteo Cataldi, Valeria Capra, Stefano Gustincich, Federico Zara, Jean-Francois Desaphy, Chiara Fiorillo

**Affiliations:** 1 Department of Pharmacy-Drug Sciences, University of Bari Aldo Moro, 70121 Bari, Italy; 2 Medical Genetics Unit, IRCCS Institute “G. Gaslini”, 80131 Genoa, Italy; andreaaccogli@ospedale-gaslini.ge.it (A.A.); micheleiacomino@ospedale-gaslini.ge.it (M.I.); 3 Department of Physics, Université de Montréal, Montréal, QC H3C 3J7, Canada; rikard.blunck@umontreal.ca; 4 Department of Biomedical Sciences and Human Oncology, School of Medicine, University of Bari Aldo Moro, 70121 Bari, Italy; concetta.altamura@uniba.it (C.A.); jeanfrancois.desaphy@uniba.it (J.-F.D.); 5 Department of Physiology and Biochemistry, Faculty of Medicine and Surgery, University of Malta, MDS-2080 Msida, Malta; cristina.dadamo@um.edu.mt; 6 Paediatric Endocrinology Unit, IRCCS Institute “G. Gaslini”, 80131 Genoa, Italy; annaallegri@ospedale-gaslini.ge.it; 7 Paediatric Neurology and Neuromuscular Disorders Unit, IRCCS Institute “G. Gaslini”, 80131 Genoa, Italy; marinapedemonte@ospedale-gaslini.ge.it (M.P.); noemibrolatti@ospedale-gaslini.ge.it (N.B.); stellavari@ospedale-gaslini.ge.it (S.V.); chiara.fiorillo@edu.unige.it (C.F.); 8 Neuropsychiatric Unit, IRCCS Institute “G. Gaslini”, 80131 Genoa, Italy; matteocataldi@ospedale-gaslini.ge.it; 9 Neurosurgery Unit, IRCCS Institute “G. Gaslini”, 80131 Genoa, Italy; valeriacapra@ospedale-gaslini.ge.it; 10 Department of Neuroscience and Brain Technologies, Istituto Italiano di Tecnologia, 80131 Genoa, Italy; stefano.gustincich@iit.it; 11 Department of Neurosciences, Rehabilitation, Ophthalmology, Genetics, Maternal and Child Health, University of Genoa, 16126 Genoa, Italy

**Keywords:** *KCNA1*, myokymia, neuromyotonia, muscle hypertrophy, ataxia, creatine kinase, patch clamp, molecular modeling

## Abstract

The *KCNA1* gene encodes the α subunit of the voltage-gated Kv1.1 potassium channel that critically regulates neuronal excitability in the central and peripheral nervous systems. Mutations in *KCNA1* have been classically associated with episodic ataxia type 1 (EA1), a movement disorder triggered by physical and emotional stress. Additional features variably reported in recent years include epilepsy, myokymia, migraine, paroxysmal dyskinesia, hyperthermia, hypomagnesemia, and cataplexy. Interestingly, a few individuals with neuromyotonia, either isolated or associated with skeletal deformities, have been reported carrying variants in the S2–S3 transmembrane segments of Kv1.1 channels in the absence of any other symptoms. Here, we have identified by whole-exome sequencing a novel de novo variant, T268K, in *KCNA1* in a boy displaying recurrent episodes of neuromyotonia, muscle hypertrophy, and skeletal deformities. Through functional analysis in heterologous cells and structural modeling, we show that the mutation, located at the extracellular end of the S3 helix, causes deleterious effects, disrupting Kv1.1 function by altering the voltage dependence of activation and kinetics of deactivation, likely due to abnormal interactions with the voltage sensor in the S4 segment. Our study supports previous evidence suggesting that specific residues within the S2 and S3 segments of Kv1.1 result in a distinctive phenotype with predominant musculoskeletal presentation.

## 1. Introduction

The *KCNA1* gene encodes the voltage-gated potassium channel Kv1.1, which regulates nerve cell repolarization after an action potential and contributes to neuronal excitability, firing rate, and neurotransmitter release [1,2,3]. The Kv1.1 channel is formed by the tetrameric assembly of four alpha subunits, each containing six transmembrane segments (S1–S6) that include functionally critical voltage-sensing and pore domains [4]. Kv1.1 dysfunction is causative of a broad range of disorders of the central and peripheral nervous systems [5,6]. Mutations in *KCNA1* have been originally associated with episodic ataxia type 1 (EA1), a rare neurological movement disorder characterized by recurrent episodes of ataxia and myokymia since early childhood [7]. These episodes are typically triggered by physical and emotional stress (including fatigue and exercise), ischemia, and changes in temperature and can last from minutes to hours. In recent years, the phenotypic spectrum has been broadened to either EA1 plus seizures [8,9] or epileptic encephalopathy (EE) [10,11], cognitive impairment, myokymia, migraine, paroxysmal kinesigenic dyskinesia, hyperthermia, hypomagnesemia, metabolic alterations, and cataplexy [12,13,14,15,16,17].

Moderate muscle hypertrophy with increased muscle tone and bilateral calf hypertrophy have also been described in some EA1 patients [6]. Furthermore, a pure musculoskeletal phenotype lacking other *KCNA1*-related features has been described in a few individuals, displaying either isolated recurrent episodes of myokymia [18] or myokymia associated with muscle hypertrophy and skeletal deformities [19,20,21]. *KCNA1* disease-causing mutations have been reported to reduce channel activity by decreasing the number of active channels in the plasma membrane or by impairing the voltage-dependent gating and kinetics of the Kv1.1 channel or both [6,22].

Here, we report a new de novo missense variant (c.803C > A (p.Thr268Lys)) in the *KCNA1* gene of a 9-year-old boy with a musculoskeletal phenotype characterized by rhabdomyolysis, lower limb stiffness, neuromyotonia, muscle hypertrophy, short stature, and skeletal deformities. The mutation T268K is located in the S3 segment belonging to the voltage sensor domain of the Kv1.1 tetramer and involves a residue that is highly conserved among other members of the Kv1 channel family. Through patch-clamp electrophysiology and molecular modelling, we studied the function of the mutant channel and elucidated the impact of the amino acid change on channel gating. Such characterization allowed us to gain insight into the genotype–phenotype correlation in the proband. Hence, our study supports the hypothesis that the functional impairment of specific residues may result in a distinctive phenotype with primarily musculoskeletal presentation.

## 2. Materials and Methods

### 2.1. Clinical and Genetic Analysis

Physical, neurological, and genetic investigations were conducted in accordance with the Declaration of Helsinki and with protocols approved by the institutional review boards of Gaslini Children’s Hospital (Protocol approval ID: 163/18; Issued on: 23/04/2018). The proband’s parents gave their informed consent before the participation in the study.

DNA was isolated from the peripheral blood by standard methods. Molecular 105 karyotyping was performed by using Human Genome CGH Microarray Kit G3 180 (Agilent 106 Technologies, Palo Alto, CA, USA) with ~13 kb overall median probe spacing. Labelling and 107 hybridization were performed following the protocols provided by the manufacturers. A 108 graphical overview was obtained using Agilent Genomic Workbench Lite Edition Software 109 6.5.0.1.

Whole-exome sequencing (WES) was performed on research basis with a trio-based approach. Genomic DNA was isolated from 1 mL of peripheral blood using the QIAamp DNA Blood Midi Kit (Qiagen). Genomic DNA was enriched with the SureSelect Clinical Research Exome 54 Mb (Agilent Technologies). The exonic sequences were captured with the xGen Exome Research Panel (IDT), and sequencing was performed on NovaSec 6500 (Illumina). Sequencing data were processed using the commercial tool CLC Genomics Workbench version 12.0 (CLCBIO, Aarhus, Denmark) for the execution of the GATK Best Practices pipeline for WES variant analysis. Removal of duplicate reads, mean coverage of coding sequence regions, alignment, and variant annotation were performed using analytical pipelines that include publicly available tools and custom scripts. We looked at nonsynonymous exonic and splicing variants with a minor allele frequency ≤0.005 in the gnomAD database. 

### 2.2. Mutagenesis and Expression of Kv1.1WT and Kv1.1T268K Channels

The T268K (c.803C > A) mutation was introduced into the plasmid PMT2LF-hKv1.1 containing the full-length human Kv1.1 wild-type cDNA using the QuikChange™ site-directed mutagenesis kit (Stratagene Cloning Systems, Santa Clara, CA, USA). The complete coding region of the cDNA was sequenced to exclude polymerase errors. HEK293 cells in a 10 mm diameter Petri dish were transiently transfected with wild-type (WT) hKv1.1 and/or the T268K variant (5 μg) and CD8 reporter plasmids (1 μg) using the calcium phosphate precipitation method. Only cells bound by anti-CD8 antibody-coated microbeads (Dynabeads M-450, Thermo Fisher Scientific) were used for patch-clamp recordings.

### 2.3. Electrophysiology

Whole-cell patch-clamp experiments were performed at room temperature (~20 °C) using an Axopatch 200B amplifier and Digidata 1550B AD/DA converter (Axon Instruments, Sunnyvale, CA, USA) [16]. Currents were low-pass filtered at 2 kHz and digitized with sampling rates of 50 kHz. The bath solution contained (mM) NaCl, 142; KCl, 2.8; MgCl_2_, 1; CaCl_2_ 1, HEPES, 10; glucose, 11; pH = 7.4, whereas the pipettes were filled with solution containing (mM) NaCl, 10; K-glutamate, 132; MgCl_2_, 2; CaCl_2_, 0.9; EGTA, 1, HEPES, 10, pH = 7.4 [16]. The pipettes were pulled from borosilicate glass and had ~2.5 MΩ resistance.

Potassium currents were evoked by 200 ms depolarizing commands from a holding potential of −80 to +20 mV (5 mV intervals). To measure tail currents, each prepulse was followed by a 150 ms voltage step at −50 or −30 mV for Kv1.1 wild-type and T268K channels, respectively. Normalized tail currents were plotted as a function of the prepulse potential, and data points fitted with the Boltzmann function I = 1/1 + exp{−(V−V_1/2_)/*k*} to determine the voltage dependence of channel activation. V_1/2_, the half-maximal activation potential, and k, the slope factor, were calculated from the fit.

To measure the kinetics of activation, currents were elicited by 200 ms depolarizing pulses from a holding potential of −80 to +40 mV (5 mV intervals). To measure the kinetics of deactivation, currents were elicited by 200 ms depolarizing pulse at +20 mV, followed by 200 ms depolarizing pulses from −80 to +20 mV (5 mV intervals). The kinetics of activation and deactivation were determined by fitting activating and deactivating current traces with a single exponential function. The calculated time constants were plotted as a function of voltage and fitted with the equation τ = τ_V1/2_ exp(V − V_1/2_)/k, where τ_V1/2_ is the time constant at the V_1/2_ of the channels, and k is the slope factor for the voltage dependence of the time constants.

To determine the slow C-type inactivation, a test pulse to +40 mV was delivered for 90 s to cells expressing wild-type and mutant channels. The kinetics of inactivation were estimated by fitting the time course of current decay with a double exponential function and calculating the fast (τ_fast_) and slow (τ_slow_) time constants and the relevant amplitudes (A %).

The recovery from the C-type inactivation was determined by using a double-pulse protocol to +20 mV, separated by interpulse intervals of increasing duration (range: 0.1–17.1 s). The peak currents evoked by the second pulse (100 ms) were divided by the first pulse (20 s), normalized, and plotted as a function of the interpulse duration. Data points were fitted with an exponential function from which the time constants were calculated.

Data were analyzed by using pCLAMP 10.3 (Axon Instruments, Sunnyvale, CA, USA) and KaleidaGraph Software. Results were reported as mean ± SEM from n cells, and statistical analysis was performed using Student’s *t*-test, with *p* < 0.05 (*) or *p* < 0.01 (**) considered as significant.

### 2.4. Homology Modeling

A homology model of Kv1.1 was built from the crystal structure of the Kv1.2/2.1 chimera (PDB 2R9R) [23]. Residues that differed or were missing in the Kv1.2/2.1 chimera as compared with the human Kv1.1 were replaced and modeled, respectively, using Modeller 9.25 [24,25]. We introduced the mutation T268K in two adjacent of the four subunits in the Kv1.1 tetramer. The resulting heterotetramer with two wild-type and two mutant subunits was introduced into a POPE: POPC: PSPS membrane (3:2:1) using CHARMM-GUI [26,27,28] and was equilibrated using NAMD 2.14 [29].

## 3. Results

### 3.1. Case Report

The proband was the second child to healthy and non-consanguineous parents. He was born at 39 weeks’ gestation following a pregnancy complicated by gestational diabetes controlled by diet. The growth parameters at birth were 3710 g weight (+0.65 SD), 50 cm length (−0.35 SD), and 35 cm head circumference (+0.18 SD). APGAR was 8–9.

On the second day after birth, he developed rhabdomyolysis with diffuse increase of muscle tone and elevated serum creatine kinase (CK) (17872 U/reference range: 15–130) in the context of febrile illness promptly treated with a broad antibiotic spectrum. Several investigations, including liquor analysis, brain MRI, and EEG, yielded normal results. This episode resolved in a few days, and CK returned to the baseline. During the second month of life, he was found to have bilateral congenital hip dysplasia that was successfully treated with the Pavlik harness for a month.

He achieved first motor developmental milestones at the expected age, despite referred abnormal gait. Speech development was normal, and there were no concerns about his cognitive functions. Since the first year of life, he experienced recurrent episodes of diffuse muscle stiffness and cramps triggered by pain or intense exercise that lasted up to 2 hours and spontaneously resolved.

**Figure 1:**
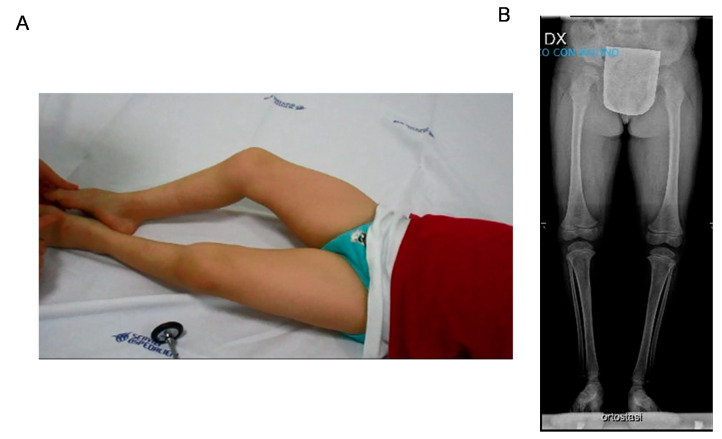
(**A**) Patient at rest aged 9 years. Lower limb stiffness with bent knees and hip posture is evident. Increased muscle bulk of tight muscles is also pictured. (**B**) X-rays of lower limbs in standing position showed shallow hip acetabulum, flattened femoral epiphyses, and slender diaphysis of the tibia.

When we first saw the patient at the age of 3 years, we noticed muscle stiffness with increased tone especially at the lower limbs without signs of pyramidal tract involvement. Deep tendon reflexes were absent with no Babinski sign. He had, however, a tiptoe ambulation with bent knees and joint retraction at the ankles. We did not notice grip or eyelid myotonia; however, he presented a coarse face, and the mother referred occasional stiffness in the mouth opening. Nerve conduction studies documented reduced motor velocities. EMG was not performed due to low compliance (Figure 1).

On physical exam, the liver was palpable 2 cm below the costal margin. The association of hepatomegaly with some coarse facial features was first suggestive of lysosomal storage disorders that were ruled out by urine dosage of glycosaminoglycans and specific dosage enzymes. Abdominal ultrasound confirmed the finding of hepatomegaly.

Brain MRI showed mild pituitary and olfactory bulb hypoplasia, but there were no other structural anomalies. Hormone levels were in the normal range. He also had normal spinal MRI, cardiac ultrasound, and ophthalmological evaluation. During childhood, he continued to experience spontaneous, self-resolving episodes of cramps and muscle stiffness, often during illness or physical stress. CK fluctuated between normal and mild increased level (≈200 U/L). Lower limb rigidity was stable, but growth was impaired. Overall phenotype was suggestive of Schwartz–Jampel syndrome, which was excluded in the genetic test.

At the last follow-up at the age of 9 years, the growth parameters were 18.6 kg weight (−2.4 SD) and 111 cm height (−3 SD). The patient presented a coarse face with mandibular prognathism. He had diffuse muscle hypertrophy, with the exception of mild distal muscle hypotrophy of the lower limbs. The lower limbs appeared bent at the knees and hips both at rest and during ambulation with the presence of tiptoeing. This posture and the tiptoe ambulation were more evident after a few jumps or running (supplementary video). There was no alteration of strength, coordination, and light touch sensation. He had kyphoscoliosis, lumbar hyperlordosis, and flat feet.

EEG showed abnormal frontal and parietal activity, especially during hyperventilation. EMG failed to reveal spontaneous discharges. X-rays of the lower limbs showed in orthostasis shallow hip acetabulum, flattened femoral epiphyses, and slender diaphysis of the tibia.

The patient started carbamazepine at a dose of 200 mg/die with initial benefit in particular on lower limb stiffness, posture, and ambulation.

### 3.2. Genetic Results

CGH-array identified a paternally inherited 1.33 Mb deletion in the chromosome 17p12 (17:14111772-15442066) according to the UCSC Genome Browser (hg19; GRChBuild 37.1, February 2009). The deletion encompasses the *PMP22* gene, whose haploinsufficiency is responsible for hereditary neuropathy with liability to pressure palsies (HNPP) (MIM 162500). This result was consistent with the finding of nerve conduction abnormalities. Of note, there was no further candidate gene in the 17p12 deletion that could explain the predominant musculoskeletal features of our patient.

From the WES output, stepwise filtering retained a de novo variant in *KCNA1* (NM_000217.3: c.803C > A (p.Thr268Lys)) that was absent in gnomAD and was predicted to have a deleterious effect by *in silico* analysis, including protein prediction and evolution conservation tools (CADD, 26.5; REVEL, 0.9917; DANN, 0.9929; SIFT, 0.9125; Polyphen, 0.992; mutation taster, 0.81; GERP, 4.9699). Sanger sequencing confirmed that this variant was heterozygous in the proband and absent in his parents.

**Figure 2:**
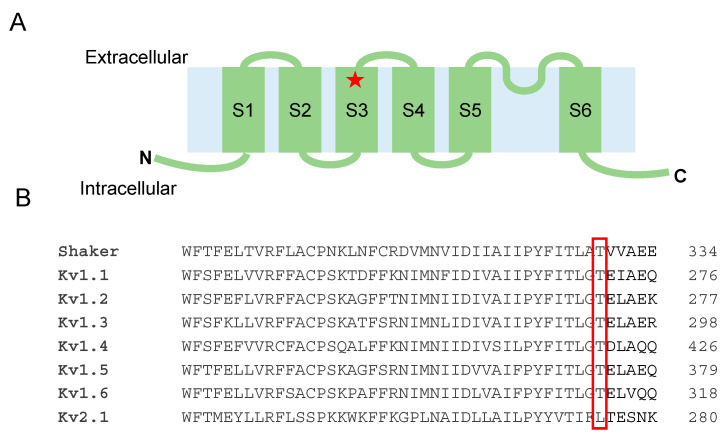
(**A**) Position of the T268K substitution in the Kv1.1 channel structure. (**B**) Amino acid alignment of Kv1 and Kv2.1 channels.

The mutation T268K is located in the S3 segment of the Kv1.1 tetramer and is highly conserved among other Kv1 channels (Figure 2).

We also identified a novel maternally inherited variant in *PHKA2* (NM_000292.3: c.3374A > T (p.Glu1125Val)) that is associated with glycogen storage disease (GSD), type IXa1/2 (MIM 306000), characterized by phosphorylase kinase deficiency in the liver, leading to hepatomegaly and hypoglycemia. Sanger sequencing confirmed that the proband was hemizygous, and the mother heterozygous for this variant. The c.3374A > T, absent in gnomAD, was predicted to be deleterious by several predictive tools (CADD, 28.1; SIFT, 0.9125; DANN, 0.9945; REVEL, 0.9779, mutation taster, 0.81); a different amino acid change was previously reported in a subject with GSD IXa1 in ClinVar (accession: SCV000823114.1). Hence, we classified this variant as likely pathogenic according to the ACMG guidelines and explained the clinical finding of hepatomegaly in our patient.

### 3.3. Functional Analysis of the Kv1.1T268K Channel

The voltage-gated K^+^ channel Kv1.1 is formed by four pore-forming α subunits. The proband was heterozygous for the disease, so he presumably possessed heterotetrameric channels composed of both wild-type and mutated subunits. To evaluate the effect of the T268K substitution on Kv1.1 function and explain symptoms in the affected patient, we expressed wild-type and T268K, either alone (5 μg) or in 1:1 ratio (5 μg + 5 μg), in HEK293 cells. The current amplitude and biophysical properties of potassium currents elicited by T268K and heteromeric WT+T268K channels were then compared with those obtained from wild-type channels.

**Figure 3:**
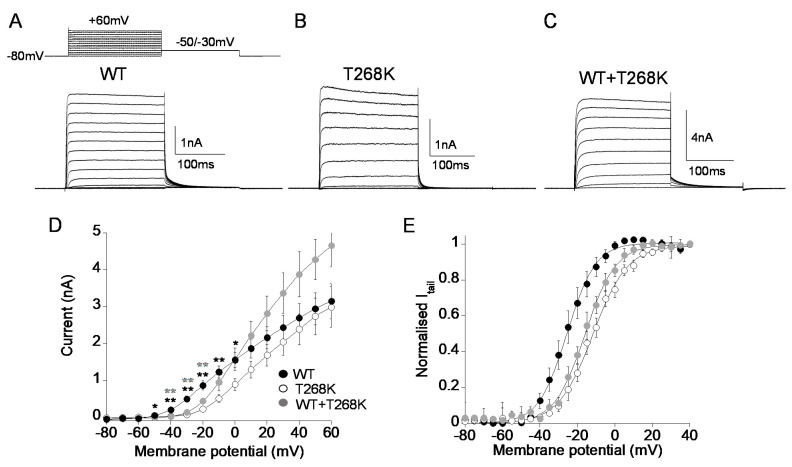
(**A**–**C**) Representative current traces evoked by 200 ms depolarizing steps from a holding potential of −80 to +60 mV from Kv1.1 WT (**A**), T268K (**B**), and WT+T268K (**C**) channels expressed in HEK293 cells. The voltage protocol is indicated in the upper panel in (**A**). (**D**) Current–voltage relationship for Kv1.1WT (5 μg), T268K (5 μg), and WT+T268K (5 μg + 5 μg) channels. (*n* = 14–36), * *p* < 0.05, and ** *p* < 0.01 with respect to WT for T268K (black) and WT+T268K (gray), respectively. (**E**) The current–voltage relationships for Kv1.1WT, T268K, and WT+T268K channels were obtained by plotting the normalized peak tail currents measured at −50 mV (for Kv1.1WT) and −30 mV (for Kv1.1T268K and for WT+T268K) as a function of the prepulse potentials and fitting data points with a Boltzmann function (*n* = 10–18 cells).

As shown in Figure 3, T268K currents measured at voltages close to the threshold for Kv1.1 channel activation (from −50 to 0 mV) were significantly smaller than those produced by wild-type channels, becoming similar to wild-type at positive potentials. Similarly, the co-expression of WT and T268K cDNAs gave rise to potassium currents significantly below those carried by wild-type channels alone from −40 mV to −20 mV. Conversely, at positive potentials WT+T268K currents were similar to the calculated sum of those carried by wild-type and mutant channels alone (Figure 3A–D). To test whether the reduction of the current levels of T268K might be due to modified voltage-dependent activation, we recorded tail currents at −50 mV for Kv1.1 and −30 mV for T268K and WT+T268K after prepulse commands to several voltages. Homomeric T268K channels shifted the voltage-dependent activation by +14 mV toward positive potentials compared with wild-type, whereas heteromeric channels resulting from the co-assembly of Kv1.1 and T268K subunits showed voltage-dependent gating intermediate between the Kv1.1 and T268K channels (Figure 3E, Table 1, Figure S1).

**Figure 4:**
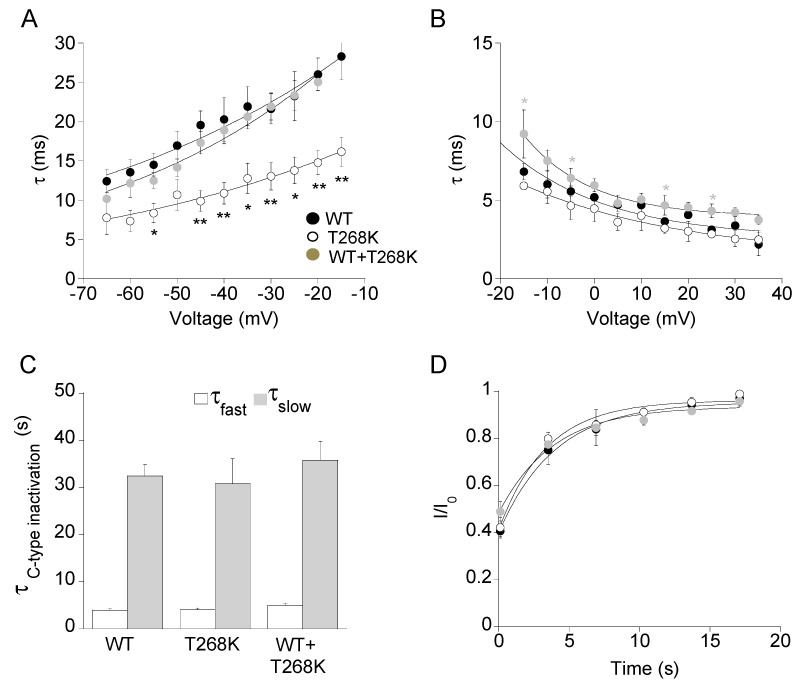
Deactivation (**A**) and activation (**B**) kinetics measured for the indicated channels. The time constants, resulting from the fit of the activating and deactivating current traces with a single exponential function, were plotted as a function of voltage (*n* = 10–19 cells), * *p* < 0.05, and ** *p* < 0.01 with respect to WT for T268K (black) and WT+T268K (gray), respectively. (**C**) Bar graphs showing the fast (white) and slow (gray) time constants of the C-type inactivation for the indicated channels calculated by fitting current decay with a double exponential function (*n* = 9–11 cells). (**D**) Recovery from C-type inactivation. The solid curves indicate the fit of the data points with an exponential function from which the time constants were calculated for the indicated channels (*n* = 6–10 cells).

To investigate whether the T268K mutation affected the kinetics of activation and deactivation of heteromeric WT+T268K channels, the activating and deactivating current traces of either wild-type, T268K, and WT+T268K channels were fitted with a single exponential function, and the calculated time constants were plotted as a function of membrane potential. As shown in Figure 4A,B, T268K channels had significantly faster kinetics of deactivation and slightly quicker kinetics of activation compared with wild-type, whereas the calculated time constants for heteromeric WT+T268K channels were more similar to those of wild-type channels (Figure 4A,B, Table 1, Figure S1).

Kv1.1 channels undergo a slow process of inactivation named C-type inactivation that develops upon tenths of seconds and affects both the duration of the action potential and the firing rate. The analysis of the slow inactivation showed that the τ_fast_ and τ_slow_ for T268K and heteromeric WT+T268K channels was not statistically different from those of wild-type channels (Figure 4C, Table 1). Accordingly, no differences were observed in the recovery from the C-type inactivation of the three channel types (Figure 4D, Table 1, Figure S1).

### 3.4. Structural Model of the Kv1.1T268K Channel

**Figure 5:**
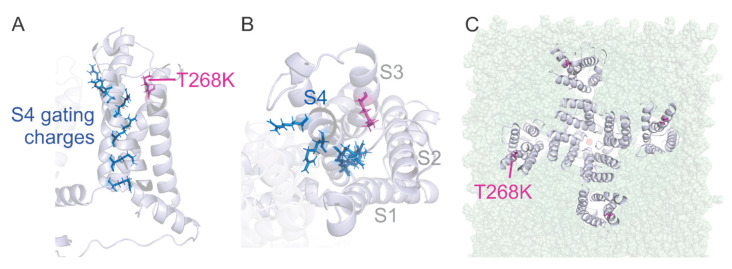
Side view (**A**) and top view (**B**) of one voltage sensor domain of the homology model of the Kv1.1 channel built upon the crystal structure of Kv1.2–Kv2.1 chimera showing the localization of the T268K mutation. T268K is highlighted in magenta and gating charges in S4 as blue sticks. (**C**) Position of the T268K mutation with respect to the lipid membrane.

To provide a molecular explanation for the electrophysiological behavior of T268K channels, we created a homology model of Kv1.1 based on the crystal structure of the Kv1.2–2.1 chimera [23] by replacing and adding residues that differed between both sequences. The homology model was placed in a phospholipid membrane and equilibrated (please refer to the Materials and Methods section for details). The position T268 is located at the C-terminus of the S3 transmembrane helix (towards the extracellular side) that belongs to the voltage sensor domain of the Kv1.1 channel (Figure 5A).

At this position, it is located at the entry to the water-filled crevice that surrounds the positively charged S4 helix (Figure 5B). This water-filled crevice concentrates the electric field around the cationic arginine and lysine residues placed at every third position in the S4 helix [30,31,32,33]. These positive charges are responsible for the voltage dependence of voltage-gated ion channels by driving the S4 helix outwards upon membrane depolarization in a combination of translation, rotation, and tilt [34]. The cationic charges are coordinated to a row of anionic charges located in S1–S3 [23] and interact with the surrounding lipid headgroups [35]. The positive charges jump along the negative charges during the conformational change [36]. The threonine at location T268 in Kv1.1 would attract water molecules and would hydrate the entry to the gating pore. While a lysine at the same position would have a similar effect, it also places a net positive charge right in the “path” of the gating charges. This adds a repulsive electrical energy to the free energy sum. To compensate for this additional energy, the membrane has to be further depolarized before the S4 can transition to its activated state. This is consistent with the shift to more depolarized potentials in the Kv1.1T268K mutant channel. The destabilization of the activated state also explains the accelerated deactivation kinetics (Figure 4B). The mutation T268K is located near the lipid headgroups, suggesting that it might interact with them too (Figure 5C).

## 4. Discussion

### 4.1. Genotype–Phenotype Correlations and Clinical Implications

Here, we describe a novel variant in *KCNA1* in a subject displaying muscle hypertrophy, neuromyotonia, and skeletal anomalies. The marked different phenotype from other EA1/EE cases and the striking analogies with the previously reported subjects with musculoskeletal features [17,18,19,20,21] both raise concerns regarding possible genotype–phenotype correlation.

Kv1.1 is composed of four α subunits, each containing six transmembrane segments (S1–S6) with S1–S4 forming the voltage sensor domain and S5–S6 the pore domain [4]. While *KCNA1* mutations associated with EA1 are spanned along the whole length of Kv1.1 [6], *KCNA1* mutations associated with epilepsy preferentially lie in specific domains of the Kv1.1 protein, such as the S1/S2 helices and the pore domain, from the end of the S4–S5 intracellular linker to the beginning of the C-terminal domain [5,8,37]. However, a genotype–phenotype correlation is challenging given the possible co-occurrence of several *KCNA1*-related features and the intrafamilial variability largely reported [38].

Regarding a specific neuromuscular phenotype linked to *KCNA1* mutation, Eunson et al. [18] first reported a 3-year-old boy presenting with an acute episode of myokymia, increased muscle tone, and elevated CK during febrile illness. He harbored the variant P244H in the S2–S3 intracellular linker that was inherited from his father, who presented with only muscle hypertrophy and subtle myokymia. Follow-up visit revealed muscle hypertrophy and tiptoe walking. Afterwards, the T226R located in the S2 segment was identified in a subject with muscle stiffness, myotonia, transient increase of CK, tiptoe walking, and multiple skeletal deformities, including knee and elbow contractions and kyphoscoliosis. Interestingly, the T226R was inherited from his mother, who showed EA without skeletal anomalies, suggesting a significant clinical heterogeneity even related to the same *KCNA1* mutation [19]. Furthermore, in the same residue, the T226K variant was found to segregate in a family with multiple affected members presenting with myokymia in the absence of episodic ataxia and epilepsy [21]. Remitting ataxia, chronic neuromyotonia with cramps, stiffness, and hypertrophy were also identified in a family carrying the L305F mutation [20].

Remarkably, our patient carrying the T268K located along the S3 segment displayed a phenotype strikingly overlapping the one of Kinali et al. [19], including multiple skeletal deformities in addition to muscle hypertrophy and stiffness and recurrent episodes of myotonia. Similarly, the predominance of musculoskeletal anomalies in our case was suggestive of Schwartz–Jampel syndrome (OMIM #255800), a rare autosomal recessive disorder caused by mutations in the perlecan gene (HSPG2, 1p34) characterized by short stature, osteochondrodysplasia, and varying degrees of myotonia. Pathogenic mutations in this gene were excluded by WES analysis, as well as mutations in other genes causing myotonia, such as *SCN4A* and *CLCN1*. Additional complexity of the patient phenotype arises from the presence of a variant in the *PHKA2* gene and the HNPP deletion. We argue that these variants can account for the liver enlargement and neurological findings, respectively, including reduced tendon reflexes, distal lower limb amyotrophy, and altered nerve conduction velocities.

Taken together, we may speculate that subjects with a specific residue change in the voltage sensor domain of the Kv1.1 channel, clustering in either S2, S3, or S2–S3 intracellular linker domains, have an increased risk to develop a primarily neuromuscular phenotype with possible associated skeletal deformities.

### 4.2. Molecular Findings and Channel Structure Implications

We performed in vitro and in silico studies to clarify the role of the T268K substitution on channel activity and structure, and gain further insight into the genotype–phenotype correlation in the proband. T268K channels expressed in HEK293 cells have faster deactivation kinetics, and their activation voltage shifted by +14 mV towards positive potentials compared with WT channels. The WT+T268K channels, which should resemble the condition of the heterozygous carrier, still require a stronger depolarization to activate. These effects imply reduced voltage sensitivity and smaller K^+^ flow at physiological voltages in agreement with functional impairment caused by other *KCNA1* mutations.

As shown from studies using the *kcna1* knock-out mouse and the Kv1.1^V408A/+^ mouse model of EA1, Kv1.1 channels, which are expressed in myelinated axons at juxtaparanodal regions and at branch points, shunt aberrant depolarization and allow proper neuromuscular transmission [39,40]. Indeed, the Kv1.1^V408A/+^ knock-in mouse revealed spontaneous bursting activity that was exacerbated by stress, fatigue, and temperature [41], whereas the knock-out mouse showed increased excitability at the sciatic nerve [42,43]. Consistently, the impaired transition to the activated state of the T268K mutant channel in our patient may render the peripheral nerve endings hyperexcitable and lead to repetitive muscle action potentials on EMG (myokymia and neuromyotonia). The proband also showed rhabdomyolysis, hypertrophy, short stature, hip dysplasia, and paraparesis. Rhabdomyolysis at birth, muscle stiffness, and hypertrophy may result from increased nerve excitability due to Kv1.1T268K channel disruption. The pathomechanism related to skeletal involvement remains to be elucidated despite the fact that a crosstalk between skeletal and neural tissues is known to be critical for bone development [44]. Fixed contractions and kyphoscoliosis may be due to prolonged and recurrent episodes of neuromyotonia, although we cannot rule out yet unknown Kv1.1 functions in the skeletal system.

The biophysical changes reported for T268K channels may result from structural rearrangements within the mutant channel. The visual inspection of the homology model of Kv1.1 carrying the T268K mutation revealed that T268 is located in the S3 segment at the entry to the gating pore. Any interaction of the C-terminal S3 and S4 with the surrounding voltage-sensing domain (S1–S2) would potentially attenuate voltage sensor movement and require additional energy to activate the voltage sensor.

The location at the entry to the gating pore is also significant. The gating pore is a water-filled crevice reaching into the voltage sensor to focus the electric field around the positive gating charges in the S4. Mutating the gating charges to histidine [30] or cysteine [31] and isolating the voltage sensor from the pore domain [33] leads to conduction through the gating pore. As we mentioned above, both threonine and lysine are of hydrophilic nature and will promote hydration of the gating pore entry (Figure 5). The positive charge of lysine at position 268, on the other hand, will additionally lead to repulsion between T268K and the gating charges in the S4 that move towards this position during gating. This would destabilize the activated state and thus require more voltage to activate the voltage sensors, explaining the positive shift in the activation voltage. A similar result has been found for an analogous mutation in the rat Kv2.1 channel, which was shifted by +16.5 mV [45]. Additionally, the Kv1.1E283K mutation in the S3–S4 linker induces a +15 mV shift in activation voltage likely due to altered interaction with S4 [16]. Like T268K, E283K is located at the entry to the gating pore. The juxtaposition of T268 to the gating charges during voltage sensor activation has been shown in the drosophila Kv1–homolog Shaker Kv. Here, the analogous position T326 can crosslink with all residues between 357 and 366 (287–296 in Kv1.1) [46]. Included are the first two gating charges, indicating that they come into close proximity (<6.5 Å) with Shaker-T326, with the consequences described above for Kv1.1T268K.

In addition to the interaction with the positive gating charges in S4, T268 is positioned to interact with the phospholipid headgroups of the surrounding membrane (Figure 5C). The charge of the lipid headgroups has been shown to alter the voltage dependence of voltage-gated potassium channels [47,48,49,50] with positively and negatively charged lipids displacing the voltage dependence to more depolarized and polarized potentials, respectively. Introducing a positive charge in this interface with T268K would interfere with the electrostatic interaction between the lipid headgroup and the gating charges, reversing the negative shift of the voltage dependence. This would also potentially explain how Kv1.1T268K shifts activation to more depolarized potentials.

Further, the analogous position to T268 in the Shaker Kv channel (T326) has been shown to influence the binding affinity of polyunsaturated fatty acids and dehydroabietic acid derivatives to the binding pocket at the voltage sensor–lipid interface [51,52]. Similarly, the introduction of a positive charge in Kv1.1 at the extracellular end of S3 might affect the binding of lipophilic small molecules to the lipid–protein cleft, opening the way to the prospective development of specific drugs to treat T268K defect.

## 5. Conclusions

**Figure 6:**
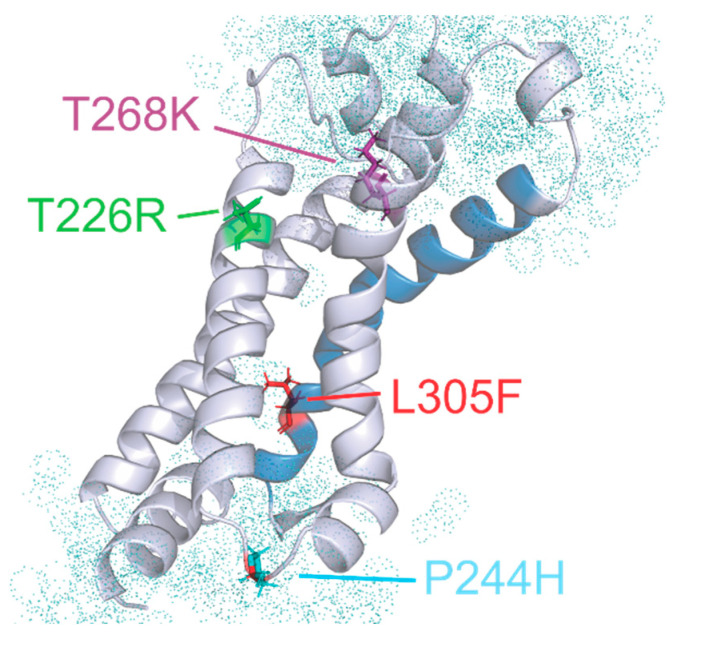
Side view of the voltage sensor domain from the homology model of Kv1.1. The pore domain, membrane, and the other three voltage sensors are removed for clarity. The S4 is shown in blue. The mutations related to neuromyotonia without significant ataxia are highlighted as stick models. Water molecules are shown as dot spheres.

We identified and characterize a novel de novo mutation in the S3 segment of Kv1.1 associated with peculiar musculoskeletal features. A similar phenotype has been reported in patients carrying other closely positioned mutations, thus supporting the hypothesis that specific residues within the S2 and S3 segments may primarily lead to myokymia, neuromyotonia, and skeletal defects (T226R, T226K, P244H, and L305F). Despite causing similar clinical features and being all positioned around the gating pore, these mutant channels show a different in vitro biophysical profile. T226R and T226K produce almost nonfunctional channels [8,21], P244H channels yield normal current amplitudes [18], and L305F possibly reduces current levels through altered voltage dependence and kinetics [20,22]. T226R was also reported to cause ataxia or epilepsy in some kindred [8,19]. So far, it is then difficult to define common structural and functional mechanisms that can lead to a clear genotype–musculoskeletal phenotype correlation. Three of the mutations alter the charge at the extracellular and cytosolic entries to the gating pore. More importantly, all of them are in positions at the interface to the lipid headgroups of the plasma membrane, making them potential targets for lipid-based therapeutics (Figure 6) [51,53,54].

## Figures and Tables

**Table 1:** Biophysical parameters of Kv1.1WT, Kv1.1T268K, and Kv1.1WT+Kv1.1T268K channels expressed in HEK293 cells.

	Voltage Dependence of Activation		Kinetic of Activation	Kinetic of Deactivation	C-Type Inactivation (Amplitude %) +40 mV		Recovery from Inactivation
	V_1/2_ (mV)	k (mV)	τ_V1/2_ (ms)	τ_V1/2_ (ms)	τ_fast_ (s)	τ_slow_ (s)	τ (s)
WT	−25.8 ± 0.4 (10)	7.4 ± 0.4 (10)	7.3 ± 0.8 (16)	25.0 ± 0.6 (10)	3.9 ± 0.3 (51%) (9)	32.4 ± 2.4 (49%) (9)	3.9 ± 0.7 (6)
**T268K**	−11.8 ± 0.3 ** (14)	9.1 ± 0.3 (14)	4.4 ± 0.8 (12)	17.0 ± 0.4 * (10)	4.0 ± 0.4 (47%) (11)	30.8 ± 5.3 (53%) (11)	3.4 ± 0.7 (8)
**WT + T268K**	−15.9 ± 0.4 * (18)	8.1 ± 0.4 (18)	5.9 ± 0.6 (19)	28.0 ± 1.0 (11)	4.8 ± 0.5 (55%) (11)	35.7 ± 4.1 (47%) (11)	3.8 ± 0.8 (10)

Data are mean ± SE. The number of cells is indicated in brackets. * *p* < 0.05, ** *p* < 0.01, with respect to WT.

## Data Availability

Data available in IRCCS G. Gaslini Children’s Hospital database.

## Supplementary Materials

The following are available online at https://www.mdpi.com/2227-9059/9/1/75/s1.
