# Supplementary material for: Musculoskeletal Features without Ataxia Associated with a Novel de novo Mutation in KCNA1 Impairing the Voltage Sensitivity of Kv1.1 Channel
Source: Biomedicines. 2021 Jan 14;9(1):75. doi: 10.3390/biomedicines9010075 (PMC7829709; doi:10.3390/biomedicines9010075)
Supplement: Supplementary file 1 [file biomedicines-09-00075-s001.pdf]

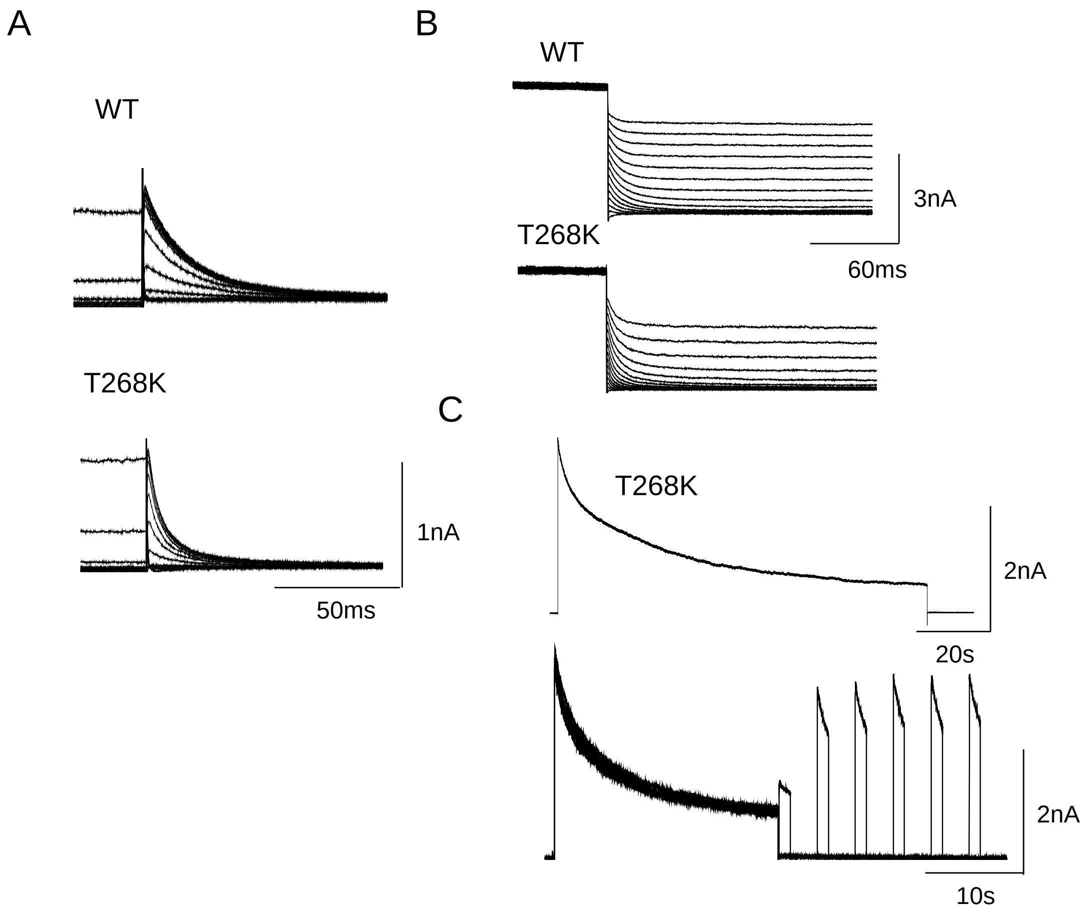

Figure S1. (A) Representative tail current families measured at -50mV for Kv1.1WT, and at -30mV for Kv1.1T68K. (B) Representative current traces showing current deactivation for the indicated channels. (C) (*Upper panel*) Sample current trace showing C-type inactivating T268K channels. (*Lower panel*) Sample current trace evoked by the two-pulse protocol for T268K channels showing recovery from C-type inactivation. Protocols are described in the Materials and Methods section.
